# Pain Genes

**DOI:** 10.1371/journal.pgen.1000086

**Published:** 2008-07-25

**Authors:** Tom Foulkes, John N. Wood

**Affiliations:** 1 Department of Stem Cell Biology and Developmental Genetics, National Institute for Medical Research, London, United Kingdom; 2 Molecular Nociception Group, University College London, London, United Kingdom; University College London, United Kingdom

## Abstract

Pain, which afflicts up to 20% of the population at any time, provides both a massive therapeutic challenge and a route to understanding mechanisms in the nervous system. Specialised sensory neurons (nociceptors) signal the existence of tissue damage to the central nervous system (CNS), where pain is represented in a complex matrix involving many CNS structures. Genetic approaches to investigating pain pathways using model organisms have identified the molecular nature of the transducers, regulatory mechanisms involved in changing neuronal activity, as well as the critical role of immune system cells in driving pain pathways. In man, mapping of human pain mutants as well as twin studies and association studies of altered pain behaviour have identified important regulators of the pain system. In turn, new drug targets for chronic pain treatment have been validated in transgenic mouse studies. Thus, genetic studies of pain pathways have complemented the traditional neuroscience approaches of electrophysiology and pharmacology to give us fresh insights into the molecular basis of pain perception.

## Introduction

Noxious environmental stimuli, tissue damage, and disease all evoke pain. The avoidance of painful stimuli, the protection of damaged tissue to promote healing, and the amelioration of disease-evoked pain (preferably through resolution of the disease itself) have obvious survival value. The evolutionary utility of pain-evoked behavioural responses is confirmed by apparent conservation of some mechanisms in all animals with nervous systems. The existence of specialised mammalian sensory neurons that respond to tissue damage (nociceptors), first proposed by Sherrington a century ago, has been clearly demonstrated in humans and mice, where mutations leading to loss of responsiveness to the trophic factor nerve growth factor (NGF) result in the loss of nociceptive neurons and a pain-free phenotype [1].

In this review we focus on genetic approaches to the mechanisms involved in activating nociceptive neurons, human genetics of pain perception, and genetic validation of pain targets leading to new drugs. The application of molecular genetics to the problem of pain has provided some remarkable insights over the past 15 years [2]. Nonetheless, the scale of the clinical problem of dysfunctional chronic pain remains vast (see for example http://www.europeanpainnetwork.com/). New drugs acting on targets validated in mouse and man by genetic studies show promise for many of the present clinical problems. This therapeutic challenge is complemented by the fact that pain also provides a wonderful model system to understand how the nervous system works. This area is perhaps the most exciting for geneticists who want to address mechanisms of perception and consciousness, neuronal signalling, synaptic plasticity, and integrative aspects of nervous system function.

## The Pain System

**Figure 1:**
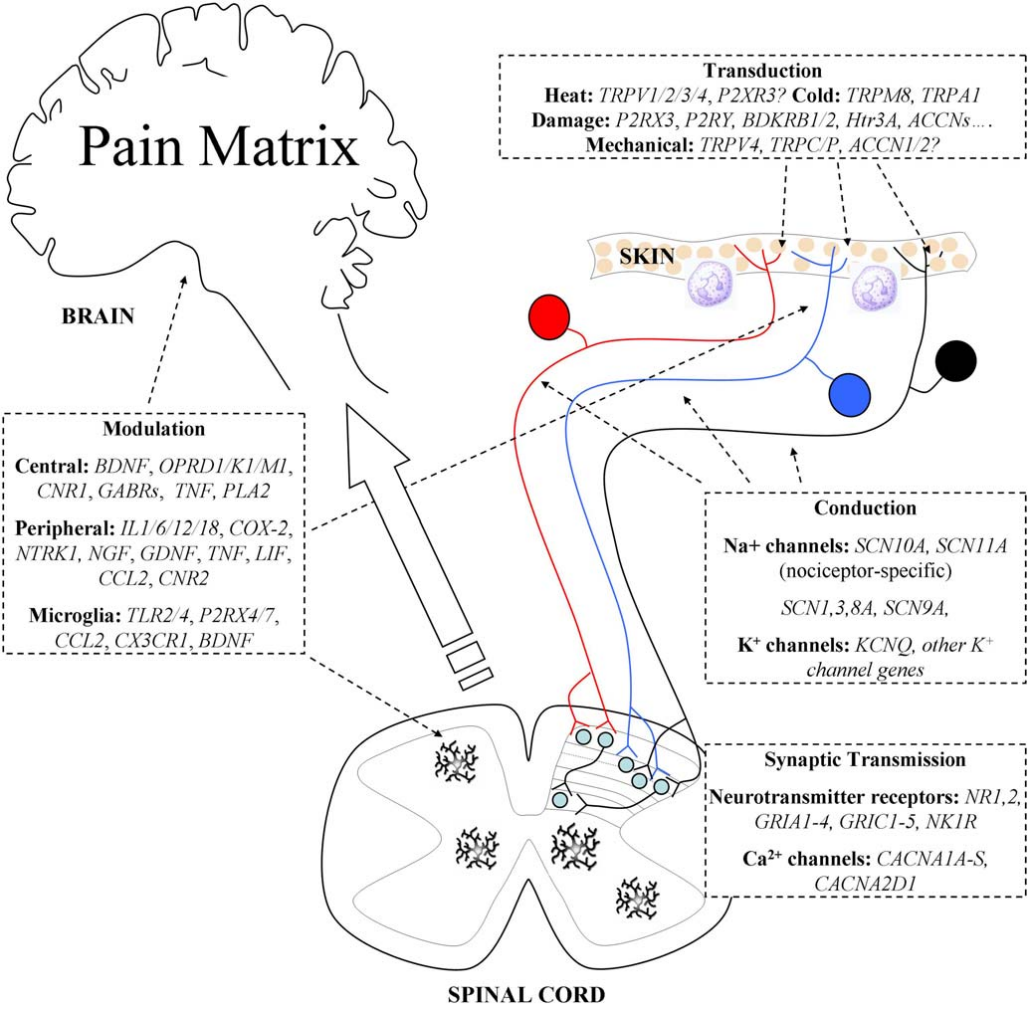
Genes Involved in Pain Perception and Modulation. Noxious stimuli are detected by primary afferent neurons innervating the skin, muscle, and viscera. The cell bodies of these damage-sensing neurons (nociceptors) are found in dorsal root ganglia, except for neurons innervating craniofacial tissues, which have cell bodies in the trigeminal ganglia. Nociceptors are commonly divided into three groups: peptidergic (blue, NGF-responsive) and nonpeptidergic (black, GDNF-responsive) unmyelinated C-fibres, and myelinated Aδ-fibres (red, NGF-responsive). Gene expression profiles differ between these groups, with functional distinctions (reviewed in [62]). Specialised receptors, expressed in the peripheral termini of these neurons, allow noxious stimuli to be transduced into electrical impulses. While the majority of nociceptors are polymodal (they can detect a variety of noxious stimuli), specific receptors exist for each stimulus modality. The receptors for certain modalities, including mechanical and heat damage, are currently unidentified, although several promising candidates have been proposed. In particular, specific TRP channels appear to be involved in transduction of several stimulus modalities (e.g., [56],[57],[63],[64]). The local membrane depolarisation generated by stimulus transduction is transmitted along the axon by voltage-gated sodium channels, some of which are expressed specifically in nociceptors. The channels Na_V_1.7 (*SCN9A*) and Na_V_1.8 (*SCN10A*) have both been shown to play an important role in nociceptive transmission [6],[7],[11]. Transmission is modulated by the actions of potassium channels, which generally act to reduce excitability. Regulation of spike frequency by potassium channels has also been reported. The sensitivity of both transduction and action potential transmission by nociceptors can be altered by inflammatory and other mediators, released by immune system cells and damaged neurons. This modulation is an important component of inflammatory hyperalgesia and neuropathic allodynia, in which neuronal sensitivity is greatly increased. Nociceptors terminate in laminae 1 (Aδ-fibres) and 2 (C-fibres) of the spinal cord dorsal horn, forming synapses with nociceptive-specific spinal projection neurons. Some also synapse with wide dynamic range projection neurons in lamina 5, while light touch neurons synapse in deeper laminae. Synaptic transmission occurs through NMDA, AMPA, and kainate receptors, in addition to neuropeptide and proton-mediated transmission. Neurotransmitter release is controlled by voltage-gated Ca^2+^ channels in the presynaptic membrane. Regulation of synaptic strength is achieved by many mechanisms, including long-term potentiation, whereby repeated activity within a frequency range results in increased response to subsequent inputs. Additionally, microglia resident in the spinal cord respond to damage or inflammation by releasing growth factors and cytokines that alter the excitability of spinal neurons. In certain cases, this can lead to the ongoing activation of pain pathways. Brain Derived Neurotrophic Factor (BDNF), fractalkine, and chemokines have been invoked as important mediators. Descending input from the brain to the spinal cord can both inhibit and facilitate the transmission of information in nociceptive circuits. From the spinal cord, information is transmitted to the brain stem via nociceptor-specific and wide dynamic range projection neurons. It is then processed by a pain matrix of multiple brain regions, resulting in both sensory-discriminative and affective pain perception ([3], reviewed in [20]). Pain pathways in the CNS are modulated by endogenous opioid peptides and arachidonic acid metabolites, acting through G-protein coupled receptors (opioid receptors and cannabinoid receptors) to limit neuronal excitability. GABAergic pathways also act to regulate the excitability of neuronal circuits involved in pain perception.

Figure 1 summarises the basic wiring and defined genes involved in the pain system. Unlike other sensory modalities, such as vision, hearing, and smell, the brain regions associated with pain perception are complex and have been best described as a pain matrix [3]. Given the uncertainty about central representation of pain sensation, it is not surprising that most research activity has focused on genes expressed by specialised peripheral sensory neurons essential for pain. The mechanisms by which noxious stimuli activate sensory neurons are summarised in Box 1. Spinal cord plasticity and descending circuits that regulate noxious input to the thalamus and higher brain centres have been examined in detail at the systems level, but genetic approaches to understanding CNS pain pathways are still at an early stage. The relationship between CNS pain perception and the activation of damage sensing neurons is complex, but most studies have focused on the more tractable aspects of peripheral pain pathways.

**Box 1. Activation of the Pain System**

Heterologous expression studies have identified a number of sensory neuron–associated genes that have been invoked as sensors for different sorts of damaging stimuli.

**Cold Pain**

Several mechanisms have been proposed for the transduction of noxious cold stimuli, including direct activation of specific TRP channels, inhibition of K^+^ currents, and reduction of electrogenic pump activity. Recent work suggests that *TRPM8* and *TRPA1* may play important roles in the detection of noxious cold, and that the inhibition of an as-yet-unidentified background K^+^ channel may be important. In addition to current research focusing on transduction by primary sensory neurons, recent reports have suggested that epidermal cells, particularly keratinocytes, may be responsible for the transduction of certain stimuli, synapsing with primary sensory neurons (see recent review from [74]). Other studies have proposed a role for vascular nociceptors in cold pain, suggesting that cold-induced vasoconstriction may begin this process [43]. It seems clear that our understanding of cold transduction is still incomplete.

The discovery that the voltage-gated sodium channel Na_V_1.8 (*SCN10A*) is not inactivated by cooling, in contrast to all other voltage-gated sodium channels, provides an insight into the mechanisms behind the detection of noxious cold [10]. It provides a clear explanation for the observation that while non-nociceptive sensory neurons become inactive upon cooling, resulting in numbness, nociceptors are sensitised. The evolutionary conservation of Na_V_1.8 reflects the vital importance of the ability to detect noxious stimuli in cold environments, particularly relevant to cold-blooded animals. A more detailed treatment of the mechanisms involved in cold pain is given in [75].

**Heat Pain**

The identification of a cation selective ion channel, *TRPV1*, expressed in sensory neurons and activated by both heat and red hot chili peppers (the capsaicin receptor), caught the public imagination and suggested that the mechanism for noxious thermosensation had been identified [76]. Despite strong evidence of a role for TRPV1 in altering inflammatory pain thresholds, the evidence that noxious heat is transduced solely or principally by TRPV1 is weak [77],[78]. Subsets of thermosensitive neurons defined by isolectin binding (IB4) have been shown not to express TRPV1 or TRPV2. TRPV1 knock-outs have fairly normal patterns of responses to acute noxious heat [77]. TRPV4 seems to have a more significant role in mechanosensation rather than thermosensation [63], but the (perhaps inappropriate) epithet thermoTRPs has been attached to these pleiotropic sensors that are involved in many aspects of sensation. It seems clear that other transducers of noxious heat, either in the skin or in neurons, remain to be identified.

**Touch and Noxious Mechanosensation**

Sensory neurons express ion channels that can be directly activated by mechanical stimuli, and evidence has been presented that such channels underlie noxious mechanosensation [79]. The genes encoding such transducing channels, however, have not been identified. Although some electrical deficits in neuronal responses to mechanical stimuli have been observed in mouse knock-outs (e.g., *ACCN3* encoding ASIC 3, as described by [80]), there are no known deficits in potential transducing channels, apart from *TRPV4* [32], that show effects on behavioural responses to noxious mechanical stimuli. Loss of sensory neurons, or downstream sodium channels characteristically expressed by these neurons (e.g, *SCN9A* and *SCN10A*), do have a mechano-insensitive phenotype [6],[11], implying that these neurons are responsible for mechanosensation.

## Peripheral Pain Pathway Neurotransmission

Tissue damage depolarises sensory neurons, but the transmission of information to the CNS requires the recruitment of voltage-gated sodium channels to propagate action potentials, and cause neurotransmitter release (mainly glutamate) into the CNS (Figure 1). In the absence of selective antagonists of ion channels, knock-out mouse studies have provided important insights into the genes that underlie this first stage in the induction of pain [4]. Three genes encoding sodium channels are selectively expressed in sensory neurons. One, *SCN11A*, is found in a subset of damage-sensing neurons, and is activated close to the resting membrane potential. Knock-out studies have confirmed that the encoded channel Na_V_1.9 does not support action potentials but plays a key role in setting pain thresholds, as it is regulated by inflammatory mediators [5]. The second, *SCN9A*, is essential for peripheral pain and had been analysed in mouse knock-outs [6] before the discovery of naturally occurring human mutants [7]. Global mouse knock-outs of *SCN9A* die, probably because they are unable to feed. However, nociceptor-specific null mutants show a loss of acute mechanical and inflammatory pain [6]. Interestingly, loss of function *SCN9A* human mutants that have a defective Na_V_1.7 channel appear to be completely pain free but are otherwise normal. This observation [7] is an important breakthrough in terms of novel target validation for new classes of sodium channel selective analgesic drugs. As an interesting corollary of this observation, human gain of function mutations of *SCN9A*, resulting in lowered thresholds of activation, result in erythermalgia, a chronic inflammatory condition. Rarer mutations that impede the inactivation of the channel seem to cause acute paroxysmal pain [8],[9]. Finally *SCN10A* (Na_V_1.8), a specific marker for nociceptive neurons, is a major contributor to electrogenesis in primary pain pathways, and is an important target for inflammatory mediators. It is also essential for cold pain [10],[11].

Regulation of sodium channel expression, both transcriptionally and posttranscriptionally, is an important element in determining neuronal excitability. A short sequence found upstream of neuronal sodium channel genes (as well as other neuronal genes) was identified and named NRSE (Neuron restricted silencing element) or RE-1 (repressor element 1) [12],[13]. Transcription factors that bound to the motif were found to act as inhibitors of gene expression in non-neuronal cells. These proteins were named REST (RE-1 silencing transcription factor), or NRSF (Neuron-restrictive silencer factor). The inhibitory activity of the complex can be further modulated by double-stranded RNA molecules that have the same sequence as NRSE/RE-1, and are found in developing neuronal precursors. These regulatory RNA molecules are able to switch the repressor function of the complex to an activator role [14]. Splicing and editing are also important regulatory elements in controlling sodium channel function. Editing events in cockroach sodium channels and *Drosophila Para* have been correlated with functional changes. Liu et al. [15] have shown that a U to C editing event resulting in a phenylalanine to serine modification can produce a sodium channel with persistent tetrodotoxin (TTX)–sensitive properties, raising the possibility that similar events could occur in mammals. Tan et al. [16] have also found that alternatively spliced transcripts can have distinct pharmacological profiles as well as altered gating characteristics.

In mammals, mutually exclusive exon usage also occurs. The *SCN3A* (Na_V_1.3) channel exists as an embryonic or adult spliced form, with different exons that code for the S3 and S4 segments in domain one of the rat channel. A similar pattern is present with *SCN9A*, where some differences in biophysical properties and the effects of cAMP on splice variants have been described [17]. A unique repertoire of sodium channel splice variants has been catalogued in DRG [18]. The presence of a transcript with a three exon repeat encoding Na_V_1.8 is enhanced by treatment with NGF, suggesting that this neurotrophin may regulate trans-splicing events in these cells [19]. All of these regulatory events are the subject of studies to assess their functional significance.

## CNS Pain Pathways and Pain Perception

Neural correlates of consciousness have been the focus of much attention, especially with the advent of functional imaging. Pain perception seems to involve a variety of discrete CNS structures [20]. Attempts to map ascending pain pathways using genetic approaches have been made [21], and there is now an appreciation of the complexity of central pain processing that involves a number of anatomically defined regulatory pathways [3]. The relationship between the level of pain perceived and the amount of input into the CNS generated by damage sensing neurons is extremely complex. The sensory–discriminatory and affective–evaluative elements that determine the nature of pain perception have been associated with distinct brain areas, but few genetic studies have attempted to address these complex issues (a study of genetic variants of *COMT* that influence the activities of endogenous opioids is discussed in Box 2). The difficulty of using genetic approaches to study central pain perception is underlined by studies from the Mogil group demonstrating empathic changes in pain thresholds in transgenic mice observing other mice in pain [22].

**Box 2. Pain-Linked SNPs in *COMT* (catechol-O-methyltransferase)**

Many SNPs affecting pain perception have been identified in recent years. Possibly the best-characterised of these are the SNPs present in *COMT*, the gene coding for catechol-O-methyltransferase (COMT). COMT mediates the inactivation of catecholamine neurotransmitters, including dopamine, adrenaline, and noradrenaline, and reduced COMT enzymatic activity appears to result in increased pain sensitivity and temporal summation of pain [26]. The precise mechanism by which this occurs has yet to be defined, although Zubieta et al. [65] propose a depletion of enkephalins due to elevated dopamine levels, leading to an upregulation of µ-opioid receptors and increased sensitivity to temporally integrated noxious stimulation. Alternatively, or perhaps additionally, decreased adrenaline metabolism (due to decreased COMT activity) may increase pain through the stimulation of β2/3-adrenergic receptors, since the pain induced by pharmacological COMT inhibition is blocked by combined β2/3-adrenergic receptor antagonists [27]. Zubieta et al. [65] investigated the effect on pain sensitivity of an abundant, nonsynonymous SNP in *COMT*. This SNP results in the substitution of valine by methionine at position 158 (val^158^met), and is associated with reduced thermostability and a 3–4-fold reduction in COMT activity. Humans homozygous for the met^158^ allele reported very slightly increased sensory and affective ratings of pain in response to muscular infusion of hypertonic saline compared to heterozygotes, while ratings for val^158^ homozygotes were lower than heterozygotes. Corresponding variations in regional, pain-related µ-opioid responses were observed using positron emission tomography, and were taken to explain the differences in pain perception [65].

A broader investigation of the influence of SNPs in the COMT gene on pain sensitivity provided a more comprehensive picture. Diatchenko et al. [26] selected six SNPs with high polymorphism frequency: two synonymous and one nonsynonymous (val^158^met) in the coding region, two in the promoter region, and one at the end of the 3′ UTR; no other SNPs exist in the coding region at frequency >0.15%. Two of these (not including val^158^met) were found to be associated with altered pain sensitivity, present in the same haploblock. The haplotype at this haploblock was found to have a significant effect on pain perception, with haplotypes designated as high, low, or intermediate pain sensitivity (population frequencies 10.7%, 36.5%, and 48.7%, respectively). This was found to correlate inversely with levels of COMT activity. Different combinations of these haplotypes were strongly associated with variations in experimental pain sensitivity. Additionally, a reduction in risk of a common painful musculoskeletal condition, temporomandibular joint disorder, was observed in the presence of even a single low-sensitivity haplotype. The mechanism by which these COMT haplotypes influence activity was recently uncovered using in silico modelling of mRNA secondary structures. It was found that the haplotypes differed with respect to mRNA local stem-loop structures, and that the most stable structure resulted in lower protein levels and thus activity. Further evidence was provided by site-directed mutagenesis, restoring normal levels of activity by eliminating the stable structure [81]. This illustrates two points: first, that synonymous polymorphisms may have large influences on protein activity; and second, the potential for interaction of multiple SNPs and therefore the importance of investigating haplotypes over single SNPs.

## Altered Pain States

Altered pain states, such as inflammatory hyperalgesia and neuropathic allodynia, are discussed in Box 3. Genetic approaches have proved valuable in revealing the mechanisms by which these states arise and are maintained.

**Box 3. Altered Pain States**

Clinical problems associated with the pain system occur when pain thresholds are dramatically altered so that noxious insults became extremely painful (hyperalgesia) or non-noxious stimuli, such as light touch or mild cold, also become very painful (allodynia). Hyperalgesia is commonly found in inflammatory pain conditions where damaged tissue is infiltrated with immune system cells, and a variety of soluble mediators that act on peripheral neurons are released. Allodynia may occur when nerves themselves are damaged—this is neuropathic pain, which occurs in situations such as diabetic neuropathy, HIV-associated neuropathy, or following physical trauma. Studies have identified a number of critical regulators of altered pain states, including genes not normally expressed in the nervous system, but present in cells of the immune system that also are activated in situations of tissue damage. For example, P2X7 (*P2RX7*), an ATP receptor absent from neurons, is essential for altered inflammatory or neuropathic pain [82]. This receptor is found on macrophages and microglia together with P2X4, and is a likely source of the soluble mediators that affect neuronal excitability in peripheral and central neurons during neuropathic pain. Damaged peripheral neurons recruit activated microglia to the dorsal horn, probably through the same mechanisms that act on peripheral immune cells, with unfortunate consequences for central pain processing [83]. A comprehensive pain database that deals with all pain-related knock-outs can be found at http://paingeneticslab.ca/4105/06_02_pain_genetics_database.asp.

Many inflammatory mediators (e.g., IL-1 and PGE2) act directly on sensory neurons and through cascades of second messengers and kinases, altering the sensitivity of both primary transducing receptors and sodium channels required for action potential transmission [84]. A pivotal role for *TRPA1* in detecting environmental irritants has recently been identified [56],[57].

## Mammalian Genetics

A wide variety of pain assays have been developed for the mouse, examining different pain modalities. Models of inflammatory and neuropathic pain are also well-described, and may have more relevance to human clinical conditions than assays of acute pain. These models have been discussed in detail by the Mogil group [23]–[25].

The variety of strains of *Mus musculus* means that the extent of genetic contributions to disease can be evaluated. For example, work on behaviour in response to various pain modalities [26],[27] identified three distinct clusters of nociception, each controlled by distinct genetic factors. Strain differences can also be used to map quantitative trait loci affecting pain thresholds.

For the investigation of the function of specific DNA sequences, transgenic and gene targeting techniques have proved useful. Examples of transgenics that have generated insights into pain pathways are numerous, and include reports of modulation of pain behaviour by the overexpression of the neuropeptide galanin [28], and the enhancement of inflammatory pain by overexpression of the NMDA receptor subunit N2RB in the forebrain [29]. Gene targeting, particularly exploiting the site-specific Cre-*loxP* recombination system to effect conditional gene deletion, has been productive [30]. Temporal control of gene expression is also possible, using inducible Cre constructs to delete *loxP*-flanked sequences upon chemical induction of Cre expression [31],[32]. Gene deletion in the mouse has proved particularly valuable to the study of pain pathways. For example, a mouse lacking *SCN10A*, the gene encoding the voltage-gated sodium channel Na_V_1.8, was recently reported to have almost complete insensitivity to cold pain [10]. The Mogil group have created a database of existing animals relevant to the study of pain [4]. This database, regularly updated and currently containing more than 230 genes, is a valuable resource to the research community. Gaveriaux-Ruff and Kieffer [33] have also catalogued neuronal Cre lines that are relevant to genetic studies of pain pathways.

## Genetics of Pain in Man

Substantial variations in both pain sensitivity and susceptibility to chronic painful conditions occur between individuals in both human and animal populations [34],[23]. For example, human pain ratings of a given thermal stimulus can encompass the entire range of the visual-analogue scale (VAS) [34], from “almost no pain” to “the worst pain imaginable.” Pain hypersensitivity may reduce quality of life and is associated with increased susceptibility to chronic pain [35]. High pain thresholds may appear desirable, but may limit protective behaviour in response to injury or hinder clinical intervention in disease.

These natural variations in propensity to pain result from a combination of environmental and genetic influences on pain-sensing systems. Environmental factors, such as early exposure to acute painful stimuli, can have long-term effects on nociceptive thresholds in both animals and humans. Perinatal painful events such as circumcision without anaesthesia, for example, have been reported to increase sensitivity to pain in later life [36], and are therefore likely to account for a proportion of the natural variability in pain perception. As discussed earlier in this review, loss- or gain-of-function genetic mutations can result in complete insensitivity to painful stimuli, or in spontaneous pain disorders (e.g., [7],[8]). It therefore appears plausible that more subtle, quantitative differences in pain sensitivity may also have a genetic basis. Gender differences in pain sensitivity have been widely reported and are discussed in Box 4.

**Box 4. Gender Differences in Pain Perception**

Gender differences in pain perception have been reported by many studies, in both animals and humans. For example, women are more likely to suffer from a variety of chronic pain disorders, including fibromyalgia, complex regional pain syndrome, and trigeminal neuralgia. Experimentally, pain thresholds for pressure pain and electrical stimulation have been shown to be lower for females than for males, while less variation has been observed for thermal pain stimuli. For a detailed treatment of gender differences in pain perception, see the recent and comprehensive review from Greenspan et al. [85]. A proportion of these variations may result from genetic differences at loci on the sex chromosomes. Gonadal factors such as testosterone and estradiol modulate sensitivity to pain and analgesia [86], resulting in gender differences in pain perception. Mogil et al. [87] reported that certain sex differences in pain and analgesia appear genetically mediated. The investigation of genetically linked factors affecting pain sensitivity, however, is confounded by contributions of gender to disease processes, and by societal influences. Nevertheless, several interesting findings have been reported, including greater opioid-induced analgesia in males than females [88],[89].

## Human Heritable Pain Conditions

Heritability studies using sensory testing of twins can identify the importance of genetic contributions to pain traits, and SNP association studies have correlated a number of genes with altered pain behaviour (see Table 1). In contrast, some rare recessive conditions found in societies that practice consanguineous marriage lead to alterations in pain thresholds, and the genes that underlie such conditions are of major interest. Selective cell loss of peripheral neurons is a characteristic of many pain insensitivity syndromes [37]. The hereditary sensory and autonomic neuropathies HSAN1-4 are all examples of syndromes where pain behaviour is diminished, and some sensory loss also occurs. Table 2 summarises currently known heritable pain conditions.

**Table 1:** SNPs Suggested To Affect Human Pain Sensitivity.

Gene	Protein	Mutation	Phenotype	Example Reference(s)
GCH1	GTP cyclohydrolase	Multiple SNPs	Partial analgesia	[35]
COMT	Catechol-O-methyltransferase	Multiple SNPs	Increased/decreased pain sensitivity	[26],[58],[65],[66]
OPRM1	Opioid receptor μ1	Multiple SNPs	Decreased pain sensitivity, decreased opioid analgesia	[52],[53]
OPRD1	Opioid receptor δ1	Multiple SNPs	Increased/decreased pain sensitivity	[67]
MC1R	Melanocortin 1 receptor	Loss of function SNPs?	Partial analgesia, increased analgesic responsiveness	[39],[68]
TRPA1	Transient receptor potential A1	Multiple SNPs	Increased pain sensitivity	[58]
TRPV1	Transient receptor potential V1	SNP	Decreased pain sensitivity	[67],[69]
CYP2D6	Cytochrome P450 2D6	Multiple SNPs	Altered analgesic efficacy	[59]
ABCB1	ATP-binding cassette, B1	SNP	Altered morphine sensitivity	[61]
FAAH	Fatty acid amide hydrolase	Multiple SNPs	Increased pain sensitivity	[58]

**Table 2:** Heritable Pain Conditions.

Syndrome	Gene Affected	Cell Loss	Phenotype	Reference
HSAN-1	Autosomal dominant mis-sense mutations in serine palmitoyltransferase long chain base subunit 1 (SPTLC1)	Apoptotic cell loss of sensory and other neurons	Pain and heat loss	[70]
HSAN-2	Mis-sense mutations in the protein kinase PRKWNK1	Developing sensory cell loss	Developing loss of all sensation	[71]
HSAN-3 (Familial dysautonomia)	Splicing deficit in IkbKAP protein	Failure in sensory neuron development	Pain-free phenotype	[72]
HSAN-4 (CIPA)	Loss of functional NGF receptor TrkA	Loss of most small diameter sensory neurons	Congenital insensitivity to pain	[1]
Mutilated foot rat	δ subunit of the (Cct4 ) gene	Loss of nociceptors	Ulceration and loss of pain sensitivity	[73]
Erythermalgia	Point mutations in sodium channel Na_V_1.7 – increased excitability	No cell loss	Chronic inflammation	[9]
Paroxysmal extreme pain (familial rectal pain)	Point mutations in Na_V_1.7 - loss of inactivation	No cell loss	Mechanically induced extreme pain	[8]
Insensitivity to pain	Mis-sense mutations in Na_V_1.7	No cell loss	Complete insensitivity to acute pain	[7]

Despite the fact that most of these syndromes involve cell death, and are thus unappealing in terms of drug development, they have provided important insights into aspects of pain signalling. For example, HSAN-3 demonstrates the essential role of NGF-dependent sensory neurons in the pain process, whilst the recent discovery of the Na_V_1.7-encoding *SCN9A* gene as an essential component of human pain provides an exciting new analgesic drug target.

## Contribution of Genetics to Pain Thresholds

Several studies have attempted to evaluate the relative contributions of genetics and environment to variations in pain sensitivity, in both animals and humans. Comparisons of pain thresholds (over 12 modalities) within and between 11 inbred laboratory mouse strains revealed genetic contributions of between 30% and 76% [23]–[25]. The experimental power of these studies was high due to the lack of environmental variability and the relatively large genetic differences between strains, meaning that these results are likely to represent the maximum contribution of genetic factors to pain perception. This contribution, however, appears to be dependent on both severity and modality of stimulus, with three major clusters of nociception identified [23],[24]. These clusters were defined as “assays of baseline thermal nociception,” “spontaneously-emitted responses to chemical stimuli,” and “baseline mechanical sensitivity and cutaneous hypersensitivity.” Surprisingly, acute thermal and mechanical nociception were found to be strongly negatively correlated [24]. While there is a body of literature supporting distinct pathways for these modalities, including different primary afferent and central neurons, differential opioid modulation, and possibly different spinal cord lamina neuron location, this would be expected to result in a lack of correlation. Negative correlation implies the presence of common pathways acting in opposing directions, or in a competitive manner. For example, a particular factor may sensitise mechanical but reduce thermal nociception, with high- and low-expressing strains resulting in the negative correlation observed. These nociceptive clusters may have a physiological basis. Sensitivity to analgesics, including gabapentin, morphine, and NSAIDS, is also affected by genetic factors in rodents [38]. For example, variations in the melanocortin-1 receptor gene have been shown to affect µ-opioid analgesia in both mice and humans [39], and *KCNJ9* (GIRK3) has been identified as a locus affecting analgesia from multiple drug classes [40].

In humans, the genetic contribution to variation in pain sensitivity has been investigated using twin studies, comparing correlation of pain thresholds between monozygotic (shared genetic and environment) and dizygotic (shared environment) twins. Two recent papers investigating responses to experimentally induced pain reported genetic contributions to sensitivity to the majority of pain modalities. Norbury et al. [41] performed quantitative sensory testing on almost 100 pairs of female twins, using a wide range of noxious stimuli and including models of hyperalgesia and allodynia. Genetic components of 22%–55% were reported for the majority of painful stimuli, particularly heat pain thresholds, including burn, burn-evoked mechanical allodynia, and iontophoresis of acid or ATP. Nielsen et al. [42] performed a similar study, investigating the genetic contribution to contact heat and cold pressor painful stimuli in a similar sample size. Sixty percent of the variance in cold pressor pain was predicted to be genetically mediated, compared with only 26% of the variance in heat pain. Interestingly, the factors influencing pain ratings were only loosely correlated between pain modalities, with genetic factors common to both modalities accounting for only 7% and 3% of the variance in cold pressor and heat pain, respectively. This is likely to be due to the nature of the cold stimulus used in this study, acting mainly through venous rather than cutaneous nociceptors [43] and therefore through a distinct neuronal pathway. Alternatively, the low correlation may reflect genetically encoded differences in primary transduction mechanisms. The differences in genetic contributions to various pain modalities in humans may reflect the distinct types of nociception described by Mogil et al. [24], probably representing peripheral or central mechanistic differences in the perception of different pain modalities. Genetic factors affecting multiple modalities of pain perception, in contrast, may represent common mechanisms in primary afferent neurons or central pain pathways.

In addition to experimentally induced pain, several studies have investigated the contribution of genetic variability to differences in severity of and susceptibility to chronic pain conditions. For example, a recent study, using 15,950 pairs of twins, found a genetic contribution of about 50% to the likelihood of developing fibromyalgia, a relatively prevalent chronic pain condition [44]. The importance of genetic contributions to chronic pain has also been illustrated using rodent models of inflammatory and neuropathic pain [45],[46].

## Mechanisms of Genetic Control of Pain Sensitivity

While these studies have been useful in defining the importance of genetic variation in differences in pain perception, they do not provide mechanistic explanations of these differences. Analysis of clinically presenting, congenital disorders of pain sensation can define genes central to the ability to detect noxious stimuli, but rely on the resulting phenotype being relatively conspicuous. Quantitative trait locus (QTL) mapping (discussed in [47]), which identifies regions of the genome associated with variation in a particular trait, is one way in which genetic factors responsible for smaller variations in pain sensitivity can be detected. This technique has been used extensively by Mogil and others to define areas of the murine genome contributing to pain sensitivity (e.g., [48],[49]). Using fine mapping, areas of <1–5 centiMorgans can be defined [47]. In addition, a number of papers analysing QTL contributions to analgesia have been published [50].

Following identification of loci associated with variability in pain sensitivity, the analysis of single nucleotide polymorphisms (SNPs) can be used to determine more precisely the genetic location underlying this variability. SNPs are traditionally defined as having an allele frequency of >1% or >0.5% in the population, although this is no longer a strict requirement. They may occur within any part of the genome: coding and noncoding sections of genes, and in intergenic regulatory or undefined regions. Coding region SNPs which do not alter the resulting amino acid are termed synonymous. Although synonymous SNPs do not alter the sequence of protein produced, they may still result in major changes in function. For example, they may affect levels of transcription, splicing, mRNA stability, or regulatory RNA expression. One mechanism by which this may occur is through effects of synonymous SNPs on cotranslational folding [51]. Sodium channels are known to be regulated by splicing and microRNAs with important functional consequences for pain thresholds. Nonsynonymous SNPs result in an altered amino acid sequence, with functional consequences that can be more directly assessed. Since genetic loci are not randomly segregated during meiosis, sets of SNPs have a tendency to be inherited together. Particular combinations of linked SNPs are described as haplotypes (from haploid genotype). This means that an identified SNP may function as a marker of another linked genetic region affecting pain processes, rather than as a mechanistic factor. Additionally, several linked SNPs may affect pain perception, meaning that haplotype analysis may be more useful than that of single SNPs for the prediction of pain sensitivity.

## Pain-Related SNPs

The study of pain-related SNPs in the human has proved problematic, due to the tendency of different data sets to yield conflicting conclusions; in many cases, a finding from one has been contradicted by those from others. Here, we present a selection of the positive associations that have been reported, with the caveat that a number of these findings have not been replicated in other investigations.

SNPs in several genes have been found to have substantial impacts on pain sensitivity. These are summarised in Table 1. Perhaps the most studied of SNPs are those in the gene *COMT*, which are discussed in Box 2. Polymorphisms in the µ-opioid receptor gene *OPRM1* (A80G, A118G), which mediates the physiological anti-pain effects of endorphins, have been linked to variations in experimental pain sensitivity [52],[53]. Interestingly, the A118G SNP in *OPRM1* (frequency ∼10%) affected both subjective pain reporting [52] and objective pain-related cortical activity [53]. Opioid analgesic requirements (representing either pain intensity or opioid efficacy) in chronic [54] pain are also affected by this polymorphism, which appears to act by down-regulating receptor mRNA [55].

The melanocortin-1-receptor gene *MC1R* contains polymorphisms associated with both pain sensitivity and µ-opioid analgesia in humans and mice [39], which also result in red hair and altered κ-opioid analgesia in females. The mechanisms behind these effects are unclear, but may involve altered µ-opioid action.

In a study going from initial pathway identification to human pain variation, Tegeder et al. [35] recently described the involvement of tetrahydrobiopterin (BH4, a cofactor in nitric oxide, serotonin, and catecholamine production) and its synthesising enzyme GTP cyclohydrolase (*GCH-1*) in pain sensitivity. Excess BH4 is thought to increase pain due to increased nitric oxide synthesis. A pain-protective haplotype (probably consisting of five SNPs in a regulatory region of the gene) of GTP cyclohydrolase was identified in about 15% of the population studied, shown to decrease both persistent post-surgical and acute mechanical experimental pain. Subjects heterozygous or homozygous for the pain-protective haplotype were found to show reduced upregulation of the *GCH1* transcript in response to cAMP, resulting in lower levels of BH4, suggesting that altered *GCH1* transcriptional modulation underlies the decreased pain sensitivity observed in these individuals.

Genetic variations in the *TRPA1* gene encoding a channel activated by cold, mustard oil, and possibly mechanical stimulation in nociceptors [56],[57], contribute to variations in cold pain sensitivity [58]. This effect was reported to be gender-dependent, in agreement with the effect of *TRPA1* deletion on cold pain in the mouse [20]. SNPs in fatty acid amide hydrolase (*FAAH*), which inactivates the endocannabinoid anandamide, may also contribute to variation in pain sensitivity [58].

## Genetic Polymorphisms Affecting Analgesia

A range of genetic variations have been identified that alter the effectiveness of analgesic drugs. In particular, polymorphisms of the cytochrome P450 enzymes (*CYP*), which play a key role in the metabolism of many drugs, can affect the efficacy of opiates, and NSAIDs. Reduced activity of cytochromes can either reduce or enhance analgesic efficacy, depending on the activity of the metabolites compared to the original drug. For example, one metabolite of the opioid tramadol, O-desmethyltramadol, is a considerably more potent agonist of the µ-opioid receptor than tramadol, meaning that low metabolisers of tramadol display reduced analgesia, despite an increased half-life of tramadol being observed [59]. Altered tramadol metabolism has been linked to polymorphisms in the gene coding for cytochrome P450 2D6 (CYP2D6), which also associate with reduced effectiveness of this analgesic [59],[60]. Polymorphisms in other cytochrome P450 isoforms also appear to contribute to variations in analgesic efficacy, generally in a drug-specific manner. Additionally, polymorphisms affecting the activity of the multidrug resistance protein ABCB1 (*MDR1*), which is a major determinant of morphine bioavailability, can alter the efficacy of morphine pain relief [61], presumably by affecting the rate at which morphine and its metabolites are removed from the cell.

Interestingly, not all genetic variations affect all pain modalities, meaning that these must be transduced by distinct pathways. The identification of genetic variations affecting propensity to pain raises the possibility of discovering new therapeutic targets for pain. By selecting those polymorphisms affecting pain sensitivity but not other processes such as cardiac function, the likelihood of identifying a specific, selective target will be enhanced.

## Future Prospects

Substantial progress has been made in pain genetics recently. Just as a massive research attack on cancer genetics in the 1980s is now paying dividends in terms of drug treatment, so the genetic description of key signalling molecules and mediators in the pain system has provided many new targets (e.g., *P2RX7*, *SCN9A*) for analgesic drugs that are under development. Chronic pain in the absence of disease is an obvious form of dysfunctional sensation and provides a useful model system for understanding other nervous system disorders (schizophrenia, autism) that involve pathological responses to external sensations. Studies of the affective component of pain perception, combined with functional imaging, genetically manipulatable animal models, and new genetic insights into pain mechanisms offer considerable hope for our understanding not only of “pain in the brain”, but other aspects of human sensation, consciousness, and behaviour.
